# Taft's Operative Dentistry.—Second Edition

**Published:** 1868-06

**Authors:** 


### BIBLIOGRAPHICAL NOTICE.

*Taft's Operative Dentistry.*—Second Edition. Published by Lindsay and Blakiston, Philadelphia. The first edition of this "Practical Treatise on Operative Dentistry" by Prof. Taft, published in 1859, has become very familiar to the dental practitioner and student, and its value universally acknowledged. It is with pleasure therefore, that we note the issue of a revised edition which the author's industry has made still more worthy of professional attention.

A careful examination of the revised edition shows us that Chapter 1, comprising articles on Calcareous Deposits, Irregularity, Atrophy, Exostosis, Denuding, Chemical Abrasion and Necrosis of the Teeth, differs but little from the same chapter in the first edition. From the well known ability of the author we had hoped for a more extended treatise on the subject of irregularity which is so closely connected with caries of the teeth, but conclude that Prof. Taft considers this as one pertaining more to mechanical than to operative dentistry. This is the case, only so far as the construction of appliances for correcting certain forms of irregularity is concerned.

In Chapter II. which contains well written articles on "Caries" we do not perceive any change, except the correction of typographical errors, and the substitution of one word or term for another having a similar meaning.

In Chapter III. which treats of the "Materials for Filling" we note the addition of an article on "Os-Artificiel" in which the efficacy of this material in protecting sensitive dentine and exposed pulps under metallic fillings is referred to. In the description of the manner of using Hill's Stopping, the author recommends, after dressing the surface of a filling of this material as perfectly as possible with instruments, the passing of a camel's hair-brush, with chloroform, to give a smooth surface and a perfect finish.

In Chapter IV. which describes the "Instruments for Filling," a description and illustration of "Scranton's Drill," and also of several varieties of excavators, are given ; and in the article on the "Manufacture of Excavators" some additional remarks are made on the forming and tempering of these instruments. In the article on "Plugging Instruments," in the same chapter, we find an addition of three pages devoted to a description and illustrations of lateral condensing instruments for use in cylinder filling, and for grinding and approximal surface fillings ; and due credit is given to Drs. Atkinson and Abbott for the great improvement in serrated plugging instruments. In the article on the "File" we find a description and illustration of "Redman's File Carrier" ; and in filing loose teeth, softened gutta-percha is recommended as being preferable to plaster for building up a wall about them.

We find in Chapter V. a description of the method of separating teeth with "wedges of wood," which is spoken of as being preferable to the more gradual pressure by means of rubber.

In Chapter VI. in the article on "Opening Cavities" an illustration of the "Enamel chisel" is added that of "Forbes gouges ;" and in that on "Cylinder filling," an additional method of preparing cylinders is given. In the same chapter we find an article on the "Mallet" in which the following favorable opinion of this instrument is expressed : "A more thorough condensation is made by the use of the mallet than is posssble by the hand alone ; greater precision of manipulation is attainable ; it is easier for the operator ; and usually less unpleasant to the patient." Illustrations of Salmon's and Redman's "Automatic Mallet Pluggers" appear in this article, and also a notice of Palmer's "plugger." Some suggestions are also added in the article on "Crystal Gold" to the manner in which a gold filling should be built up so as to restore the form of the crown ; and illustrations of different forms of burnishers.

Also in Chapter VII. some additional suggestions on forming cavities in the filling of certain classes of cavities ; and illustrations of a common "Saliva Pump," "Dibbles Saliva Pump," a "Tongue and Duct Compressor," and the author's "Extension Thimble." A description of the

Rubber, or Coffer Dam " and the manner of applying it is also given, and the application of os-artificiel as a substance to prevent gold from being visible through a thin wall of enamel; together with the method of restoring a portion of the cutting edge of an incisor tooth; and for approaching posterior approximal cavities of molar teeth, by cutting through the grinding surface instead of filing out a V shaped space from the approximal surfaces.

In Chapter VIII. referring to the agents used in the treatment of sensitive dentine, tannin in solution with creosote and glycerine is mentioned as a preparation recently brought into use; and the caution given that contact of chloride of zinc be permitted no further than its action is desired.

In Chapter IX on "Exposed Pulps" chromic acid is added to the escharotics recommended for a morbid condition of the pulp, especially where there is a tendency to prurient enlargement; and the systemic condition of the patient spoken of as exercising a great modifying influence upon the treatment. Dr. Allport's method of treating exposed pulps, by excision, is also given; and in the article on "Destruction of the Pulp," the objectionable method of forcing into the canal a piece of wood is omitted; also the use of chlor. sodium as a deodorizing agent, which is recommended in the first edition. In the article on "Preparing the Teeth and Roots for Filling," illustrations of Palmer's Nerve Instruments, for preparing and filling nerve canals, are given. The method of filling around a wire introduced into the pulp cavity, the wire being afterwards withdrawn, in order to leave a vent for the escape of any secretion is commented on in this edition, as always to be depreciated and only to be used as a last resort.

In the same chapter we also note the addition of an article on "Dental Periostitis" in which only the "principles involved in the treatment" are considered, not "details for special cases."

Both systemic and local treatment are recommended, and the tinct. of iodine and cantharides, the latter in the form of a preparation known as "cantharidal collodion," are highly spoken of as counter-irritants; the cantharidal collodion "acts promptly and efficiently in almost every case of acute dental periostitis." The hyperdermic injection of a solution of morphine, or tinct. of opium (ten to twenty drops) is said to give promise of most desirable results. In the article on "Alveolar Abscess," we find little or no change from that in the first edition.

In Chapter X. devoted to "Pivot Teeth," in regard to the grinding of the artificial crown to fit the natural root, or leaving it untouched by the emery-wheel, Dr. Palmer's suggestion of grinding the entire labial surface is referred to in the article on "Fitting the Crown;" and os-artificiel is recommended as being preferable to Hill's stopping in one of the methods given for attaching the pivot to the root.

In Chapter XII. which treats of the "Accidents in the Extraction of Teeth," among the styptics spoken of as being of service in arresting hemorrhage, a solution of tannin with creosote is recommended; but we find no mention made of those valuable preparations of iron, known as Monsel's Solution of the Persulphate and the Powdered Subsulphate.

In the article on the "Removal of a Wrong Tooth," the author, in speaking of the propriety of replacing detached teeth in their cavities, says that many instances have come under his observation where teeth so treated have become as firmly fixed in their sockets as before removal, and remained in an apparently healthy condition.

To Chapter XIII. on "Anæsthetics," is added a brief article on "Nitrous Oxide Gas;" also in the article on "Local Anæsthesia," Dr. Richardson's process is referred to, with a description and illustration of the "Spray Apparatus." An addition of forty-seven pages and six illustrations has been made to this edition, which is printed on better paper and in style superior to that of the first edition.

After a careful review of the revised edition we consider it as being a very creditable contribution to the literature of the specialty to which it is devoted.

